# We have the evidence but governments must now build the systems to deliver on hand hygiene

**DOI:** 10.1136/bmjgh-2025-018930

**Published:** 2025-09-16

**Authors:** Joanna Esteves Mills, Bruce Gordon, Lindsay Denny, Ann Thomas, Arnold Oredola Cole, Andrea Lee-Llacer, Sophie Hickling, Alemu Kejela Ababu, Akosua Kwakye, Alyaa Mohamed, Aarin Palomares, Nathaniel Paynter, Om Prasad-Gautam, Siddhi Shrestha, Precious Kalubula, Bethany A Caruso, Matthew C Freeman, Marlene K Wolfe, Sudan Raj Panthi, Marysol Astrea Liwanag Balane, Jon Michael Villasenor, Kidist Bartolomeus, Anthony Eshofonie, Bonifacio Magtibay, Sory Bouare, Doussou Doumbia, Kachusha Nkosha, Rajit Ojha, Suzzy Abaidoo, Waltaji Kutane, Mahamane Toure, Issaka Sangare, Sanjani Limbu, Ahammadul Kabir, Seema Ranjouria, Doreen Mulemba, Suzan Alzubaidy, Olga Kokshagina, Leah Heiss, Elisabetta Minelli, Charles Nachinab, Oliver Cumming

**Affiliations:** 1 Water, Sanitation, Hygiene and Health Unit, Department of Environment, Climate Change and Health, World Health Organization, Geneva, Switzerland; 2 UNICEF, New York, New York, USA; 3 UNICEF East and Southern Africa Regional Office, Nairobi, Kenya; 4 Government of the Philippines, Manila, Philippines; 5 WaterAid United Kingdom Office, London, UK; 6 WASH and Environmental Health, Government of Ethiopia, Addis Ababa, Ethiopia; 7 WASH and Environmental Health, Ethiopia Ministry of Health, Addis Ababa, Ethiopia; 8 World Health Organization Country Office for Ghana, Accra, Ghana; 9 World Health Organization Country Office for Iraq, Baghdad, Iraq; 10 Global Handwashing Partnership, Washington, Washington, USA; 11 UNICEF Nepal Country Office, Lalitpur, Nepal; 12 Health Emergencies, World Health Organization, Geneva, Zambia; 13 Public Health, Zambia Ministry of Health, Lusaka, Zambia; 14 HUbert Department of Global Health, Emory University, Atlanta, Georgia, USA; 15 Gangarosa Department of Environmental Health, Emory Univ, Atlanta, Georgia, USA; 16 Environmental Health, Emory University Rollins School of Public Health, Atlanta, Georgia, USA; 17 Environmental Health, World Health Organization, Kathmandu, Nepal; 18 UNICEF Philippines Country Office, Manila, Philippines; 19 UNICEF Philippines Country Office, Mandaluyong, Philippines; 20 Product Design and Impact Unit, Science Division, World Health Organization, Geneva, Switzerland; 21 World Health Organization Country Office for Bangladesh, Dhaka, Bangladesh; 22 World Health Organization Country Office for Philippines, Manila, Philippines; 23 World Health Organization Country Office for Mali, Bamako, Mali; 24 National Health Directorate, Government of Mali, Bamako, Mali; 25 WaterAid Zambia Country Office, Lusaka, Zambia; 26 Ministry of Water Supply, Government of Nepal, Kathmandu, Nepal; 27 Ministry of Sanitation and Water Resources, Government of Ghana, Accra, Ghana; 28 World Health Organization Country Office for Ethiopia, Addis Ababa, Ethiopia; 29 WaterAid Mali Country Office, Bamako, Mali; 30 WaterAid Nepal Country Office, Kathamandu, Nepal; 31 WaterAid Nepal Country Office, Kathmandu, Nepal; 32 Ministry of Water and Sanitation, Government of Zambia, Lusaka, Zambia; 33 Ministry of Health, Government of Iraq, Baghdad, Iraq; 34 University of Sydney, Sydney, New South Wales, Australia; 35 Monash University, Melbourne, Victoria, Australia; 36 UNICEF Ghana Country Office, Accra, Ghana; 37 London School of Hygiene and Tropical Medicine, London, UK; 38 Department of Disease Control, Environmental Health Group, London School of Hygiene & Tropical Medicine, London, UK

**Keywords:** Public Health, Hygiene, Respiratory infections, Global Health, Health policy

**Summary box**

- Hand hygiene protects health, strengthening community resilience and contributing to more robust health systems.
- Strategies to improve hand hygiene should prioritise access to water and soap or alcohol-based handrub.
- Beyond project-based approaches and short-term service delivery, sustained government-led efforts to strengthen national and local systems are now needed.
- WHO and UNICEF Guidelines on Hand Hygiene in Community Settings will provide evidence-based guidance to support government-led action on hand hygiene.
- Examples of effective government leadership that takes this guidance to action are needed to inspire the global change needed.

The simple act of cleaning hands can save lives and reduce illness by helping prevent the spread of infectious diseases. Hand hygiene can lower the risk of diarrhoea and acute respiratory infections—two leading causes of morbidity and mortality worldwide—by 30% and 17%, respectively.^1 2^ Additional benefits include reducing skin and eye infections such as trachoma and intestinal worm infections such as hookworm and ascaris, which together contribute significantly to the disease burden in low-income and middle-income countries.

Hand hygiene protects health, strengthens community resilience and contributes to more robust health systems. For example, by reducing the pressure on the health system that treats infectious diseases, hand hygiene can save resources for other health priorities. It can also reduce the need for antibiotic treatment, thereby reducing the spread of antimicrobial resistance and the associated deaths and health costs.

The importance of hand hygiene to human development, emergency response and health emergency preparedness is also internationally recognised. In particular, Sustainable Development Goal 6.2 commits countries to achieving by 2030 universal access to a basic handwashing facility (one with soap and water present) in the home.^3^ The 2005 International Health Regulations^4^ and the 2025 Pandemic Prevention, Preparedness and Response Accord^5^ are two legally binding frameworks designed to curb the spread of disease globally, and which also require countries to bolster water, sanitation and hygiene services.

Despite international recognition of its importance, global progress on hand hygiene has consistently failed to measure up to political commitments and pledges. There have been gains—between 2015 and 2024, 1.6 billion people gained access to a basic handwashing facility^6^—but in 2024 1.7 billion people still lacked a handwashing facility with soap and water at home and 611 million had no handwashing facility at all.^6^ Nearly half lived in sub-Saharan Africa, a quarter in Central and Southern Asia and three out of five lived in rural areas.^6^ Achieving universal access by 2030 would require a doubling globally in current rates of progress, rising to 11-fold in least developed countries and eightfold in fragile contexts. Meanwhile, each year, 740 000 people die of diarrhoea or acute respiratory infections that could have been prevented with hand hygiene.^7^

With the rising risk of disease outbreaks and health emergencies worldwide, now is a critical moment to strengthen community resilience against infectious diseases. This can be achieved through comprehensive prevention and control measures—including hand hygiene. While many stakeholders have a role to play, overall responsibility lies with governments, through their duty to advance the individual human right to health and protect public health, and through global health security obligations.

New WHO and UNICEF Guidelines on Hand Hygiene in Community Settings expected later this year can support government-led action on hand hygiene. The guidelines provide guidance on how to improve hand hygiene, reflecting the best available evidence, published in the five systematic reviews in this supplement.^8^,^12^ This commentary has been written by experts and government leaders who have engaged in the process to develop the guidance.

Here, we summarise the evidence-based guidance, but reiterate the need to move beyond it—towards government-led efforts to strengthen national and local systems for hand hygiene.

## We know what is needed

Strategies to change and/or sustain hand hygiene behaviour should address three core requirements for hand hygiene. Like all human behaviour, hand hygiene is determined by interconnected cognitive, psychological, sociocultural and environmental factors that are highly context-specific, making it challenging to provide global guidance. However, the systematic reviews in this supplement point to three core requirements to change and/or sustain hand hygiene practice that are universally applicable. These are as follows:

Access to soap and water or alcohol-based handrub (ABHR): Without these minimum material needs, hand hygiene simply cannot be practised. This is an obvious but important point, as interventions often promote hand hygiene through information, education and marketing but without ensuring access to soap and water or ABHR. Access to these minimum material needs should be any government’s first priority.

Access to information on why, when and how to clean hands: Effective communication of vital information on hand hygiene is another necessary precondition. If people do not know why, when or how to practise hand hygiene, they may not prioritise it, may miss key times or may use materials or techniques that do not sufficiently remove pathogens from hands.

Access to a conducive physical and social environment: Access to basic materials and information alone may not be sufficient to change and sustain hand hygiene practices. A conducive physical and social environment refers to aspects of the environment that can encourage and motivate sustained practice. These aspects broadly comprise convenience, attractiveness and ease of use of hand hygiene facilities, and supportive social norms.

## Systems-based approaches can create sustainable change

Efforts to improve hand hygiene must move beyond project-based approaches and short-term service delivery, towards government-led strengthening of national and local systems for hand hygiene. Governments and international institutions often mobilise rapidly during disease outbreaks, deploying resources, strengthening health systems and raising public awareness. However, once an emergency is contained, momentum is often lost, budgets are cut, preparedness plans go dormant, and political attention shifts elsewhere. This reactive approach undermines long-term resilience, leaving systems vulnerable when the next crisis arises. To break this cycle of panic and neglect, governments should strengthen systems that can deliver hand hygiene services, as part of broader health systems.

Effective government measures to strengthen systems include providing policy and legal frameworks, regulation and monitoring that support planning and actions, and coordinating these processes through national institutions with a clear delineation of mandates and sufficient human and financial resources. These measures should enable effective, equitable and sustainable delivery of the preconditions and enabling environment needed for the sustained provision of hand hygiene services.^8 13^ These services, in turn, provide reliable, accessible and affordable hand hygiene facilities for all, along with their operation and maintenance, and ongoing behaviour change strategies for sustained practice.

## Strong leadership and investment from policy-makers are urgently needed to drive forward this agenda

Short political and funding timelines, low public and media attention outside emergencies, and competing priorities can mean that hand hygiene—and other preventive measures—are overlooked until there is an emergency.

Strong leadership from policy-makers can change this pattern, driving sustainable change. This includes setting out an inspirational vision for hand hygiene and a financed plan for achieving it and providing ongoing coordination and support to the implementation agenda.

Most importantly, political leadership requires sufficient investment to deliver change. Although cost-effective and relatively simple, hand hygiene interventions are not always low-cost. In particular, water supply infrastructure requires investment. Governments should not rely on emergency budgets, embedding hand hygiene financing instead in annual health budgets.

## Conclusions

As governments consider their commitments under the new Pandemic Prevention Preparedness and Response Accord, sustained acceleration of hand hygiene as part of broader efforts is urgent. Evidence-based guidance to support these efforts will be available in the forthcoming WHO and UNICEF Guidelines on Hand Hygiene in Community Settings. What is needed now is examples of effective government leadership that uses this guidance for action and change. 10 governments, represented with authors on this paper, have committed to adopt the new guidelines and work towards the implementation of the recommendations. The actions—and learning—of these governments can inspire and guide other governments to take action on this critical area of public health.

## Data Availability

Data sharing was not necessary for this article.

## References

[1] Wolf J, Hubbard S, Brauer M (2022). Effectiveness of interventions to improve drinking water, sanitation, and handwashing with soap on risk of diarrhoeal disease in children in low-income and middle-income settings: a systematic review and meta-analysis. The Lancet.

[2] Ross I, Bick S, Ayieko P (2023). Effectiveness of handwashing with soap for preventing acute respiratory infections in low-income and middle-income countries: a systematic review and meta-analysis. The Lancet.

[3] United Nations Department of Economic and Social Affairs. Sustainable development goals - United Nations The 17 goals. https://sdgs.un.org/goals.

[4] World health organization (2008). International health regulations (2005).

[5] World health organization (2025). Intergovernmental negotiating body to draft and negotiate a who convention, agreement or other international instrument on pandemic prevention, preparedness and response - report by the director-general. https://apps.who.int/gb/ebwha/pdf_files/WHA78/A78_10-en.pdf.

[6] United Nations Children’s Fund, World Health Organization (2025). Progress on household drinking water, sanitation and hygiene 2000-2024: special focus on inequalities. https://www.who.int/publications/m/item/progress-on-household-drinking-water--sanitation-and-hygiene-2000-2024--special-focus-on-inequalities.

[7] World Health Organization (2023). Burden of disease attributable to unsafe drinking-water, sanitation and hygiene, 2019 update.

[8] Hilton SP, An NH, O’Brien LA (2025). Efficacy and effectiveness of hand hygiene-related practices used in community settings for removal of organisms from hands: a systematic review. BMJ Glob Health.

[9] O’Brien LA, Files K, Snyder JS (2025). Minimum material requirements for hand hygiene in community settings: a systematic review. BMJ Glob Health.

[10] Caruso BA, Snyder JS, O’Brien LA (2025). Behavioural factors influencing hand hygiene practices across domestic, institutional and public community settings: a systematic review and qualitative meta-synthesis. BMJ Glob Health.

[11] Prasad SK, Snyder JS, LaFon E (2025). Interventions to improve hand hygiene in community settings: a systematic review of theories, barriers and enablers, behaviour change techniques and hand hygiene station design features. BMJ Glob Health.

[12] Snyder JS, Canda E, Honeycutt JC (2025). Effectiveness of measures taken by governments to support hand hygiene in community settings: a systematic review. BMJ Glob Health.

[13] Freeman MC, Crocker J, Chipungu J (2025). Systems thinking for hygiene in settings with high risk of infectious disease transmission. Nat Water.

